# Non-invasive Assessment of Vaccine-Induced HPV Antibodies via First-Void Urine

**DOI:** 10.3389/fimmu.2020.01657

**Published:** 2020-08-05

**Authors:** Jade Pattyn, Severien Van Keer, Laura Téblick, Pierre Van Damme, Alex Vorsters

**Affiliations:** Faculty of Medicine and Health Sciences, Centre for the Evaluation of Vaccination, Vaccine and Infectious Disease Institute (VAXINFECTIO), University of Antwerp, Antwerp, Belgium

**Keywords:** humaan papillomavirus, HPV vaccination, HPV antibodies, urine, HPV serology

## Abstract

The potential of first-void (FV) urine as a non-invasive method to monitor human papillomavirus (HPV) vaccination has been reported, mainly focusing on urine as a sample to assess HPV DNA. Besides HPV DNA, vaccine-induced HPV antibodies originating from cervicovaginal secretions were recently shown to be detectable in FV urine as well. This presents a novel opportunity for non-invasive sampling to monitor HPV antibody status in women participating in large epidemiological studies and HPV vaccine trials. The simultaneous assessment of both HPV infection and immunogenicity on a non-invasive, readily obtained sample is particularly attractive.

## Introduction

The evaluation of immunogenicity in HPV vaccine trials relies largely on serology. In the absence of a correlate of protection, it is generally accepted that the presence of high concentrations of vaccine-induced antibodies in serum—greater than those elicited by natural infection—are the best indicators of long-term protection against HPV infection (1). Nevertheless, as cervical cancers typically occur at the cervical transformation zone, it is believed that the presence of mucosal HPV antibodies at the cervix, the site of infection, is critical for vaccine-induced protective immunity. Unlike other mucosal secretions where immunoglobulin A (IgA) predominates, cervicovaginal secretions (CVS) mainly contain IgG transudate from serum and fewer locally produced IgG and secretory IgA (sIgA) (2). Hence, vaccine-induced circulating antibodies are thought to reach the site of infection by transudation at the female genital tract, and by passive exudation at sites of trauma (3). The presence of HPV antibodies at the cervix, using cervicovaginal secretions (CVS) as a proxy, has been reported in a number of studies (4–9) [reviewed in (10)], and just recently in FV urine as well (11, 12). Comparable with CVS results (9), there was an approximate 2-log difference in HPV antibody levels between first-void urine and serum (4) and moderate to good correlations between HPV antibody levels in serum and first-void urine were observed (11, 12).

## Rationale To Detect Vaccine-Induced HPV Antibodies in First-Void Urine

The presence of HPV-related biomarkers (e.g., HPV DNA) in the urine of women is based on the fact that discharged mucus and debris from exfoliated cells from the female genital organs (including the cervix) accumulate around the urethra opening, between the small labia, and are washed away with the urine flow. Consequently, the initial flow of urine—defined as first-void urine—contains significantly more human and HPV DNA than random or mid-stream urine (13–16). Currently, there remains some confusion regarding the definition of first-void urine, which should refer to the initial stream of urine but is sometimes defined as the first urine of the day. Next to the use of first-void urine, other keynotes for improved HPV DNA detection in FV urine were recently summarized (17). Rwanda and Bhutan were the first countries to show the impact of HPV vaccination using optimized urinary HPV DNA testing, confirming the relevance of this sample as representative of the genital tract. These studies also confirmed that FV urine sampling can be successfully implemented in a large cohort study of young adolescent girls (18, 19).

Standardized and optimized protocols (including collection, storage, and processing of urine samples) have significantly enhanced the sensitivity of urinary HPV detection and demonstrated good concordance with cervical samples (16, 20, 21). Furthermore, recent studies indicated that CIN2+ detection using HPV testing of urine shows a sensitivity similar to that of clinician-taken smears or brush-based self-samples (16, 20). Because of these promising results, our hypothesis was that CVS—containing HPV-specific antibodies transudated from the circular system—flushed away by the initial urine flow would also harbor HPV-specific antibodies. Recently, a proof of concept study confirmed this hypothesis, and hence the presence of measurable HPV-specific antibodies originating from CVS in FV urine (11, 12). In addition, correlations with HPV vaccination status and paired serum samples were found using two different HPV immunoassays not yet specifically designed for urine samples (11, 12), making further improvements on the accuracy level possible.

## Analyzing HPV Antibodies in First-Void Urine: Benefits, Drawbacks and Challenges

Using urine has several advantages over other more invasive sampling methods. If successful, non-invasive urine sampling could partly replace serum for follow-up of HPV-vaccination and could potentially enhance participation in vaccine trials. Moreover, as urine sampling is non-invasive and does not require trained medical personnel for collection, urine could be of great value for multiple collections at different time points (at home). From the logistical point of view, the possibility to measure both virologic (HPV DNA) and immunological (HPV antibody) end-points in FV urine could ease future surveys and provide major logistical and financial benefits. For example, expansion of HPV vaccination to lower-income countries, particularly with one-or two-dose schedules, may be facilitated by the use of such a tool to assess (early) impact (22). It is clear that, if successful, urinary HPV antibody detection could provide new possibilities for research in settings where serum samples cannot be taken and/or (self-collected) vaginal swabs are less preferred due to for example cultural reasons or a fear of discomfort.

Besides clear benefits, urine also has drawbacks. Because of inter-individual and intra-individual variability, standardization and normalization is essential. The major variations in urinary antibody concentration are determined by the amount of genital secretions washed away by the initial stream of urine, the volume of urine collected (dilution), and the amount of mucus. We are currently investigating the potential of total IgG concentration, total protein concentration, and other potential normalization markers to develop a normalization algorithm. Besides, more research is necessary to investigate the most appropriate buffer to preserve antibodies in first-void and to determine the maximum time between sample collection and detection.

Another challenge using urine consists of generating quantifiable levels of antibodies. Both the amount of antibodies at these mucosal surfaces as well as their presence in urine might be a variable. At the moment we are improving IgG concentration and isolation by pre-treating our urine samples. HPV antibody levels in FV urine were indeed around the detection limit for both the multiplex L1/L2 virus-like particles (VLP)-based ELISA (M4ELISA) and glutathione S-transferase (GST)-L1-based immunoassay (GST-L1-MIA) immunoassay, which complicates the distinction between uronegative and low positive results (11, 12). As formal non-inferiority compared to serum is the goal, there is a need for a robust HPV immunoassay that would reach reasonable high sensitivity for urinary HPV antibodies testing. Besides, research to determine the cut-off necessary to make useful conclusions from urine testing has to be established.

Our current research is focused on HPV, however, the idea and technology could be applied to any infection or disease for which monitoring (vaccine-induced) antibody responses is imperative for disease control. Hence, the use of HPV vaccine-induced antibodies could be a model for wider use of FV urine in immunoassays. It is notable, however, that this simplified sample collection is only applicable in females. Although females are a large part of the population, for most of the vaccination trials, both males and females would need to be surveyed. Hence, further research is necessary to develop accurate and feasible non-invasive sampling that can be performed by men. Oral fluid may be such possible non-invasive alternative to serum for HPV antibody surveillance in men and women (23–26).

## Concluding Remarks

It is clear that the successful development of a technology that measures antibodies in female urine as a way to determine HPV vaccination status, besides HPV DNA status, is very promising. Since FV urine collection is less invasive than serum collection, and does not require trained personnel, the validation of such procedure would be of great value for the international HPV research community. In different settings, FV urine could allow vaccine effectiveness and coverage data to be reliably generated post-implementation.

## Data Availability Statement

The original contributions presented in the study are included in the article, further inquiries can be directed to the corresponding author/s.

## Author Contributions

All authors listed have made a substantial, direct and intellectual contribution to the work, and approved it for publication.

## Conflict of Interest

PV and AV are co-founders and former board members of Novosanis (Belgium), a spin-off company of the University of Antwerp that produces a device for collection and preservation of first void urine. The remaining authors declare that the research was conducted in the absence of any commercial or financial relationships that could be construed as a potential conflict of interest.
